# Echo-Doppler Predictors of Residual Pulmonary Hypertension After Pulmonary Thromboendarterectomy

**DOI:** 10.3390/jcm14165705

**Published:** 2025-08-12

**Authors:** Estefania Oliveros, Anil Jonnalagadda, Rylie Pietrowicz, Madeline Mauri, Huaqing Zhao, Rohit Maruthi, Hollie Saunders, Vladimir Lakhter, Yevgeniy Brailovsky, Riyaz Bashir, Ahmed Sadek, Anjali Vaidya, Paul Forfia

**Affiliations:** 1 CTEPH Program, Department of Medicine, Division of Cardiology, Pulmonary Hypertension, Right Heart Failure, Temple University Hospital, Philadelphia, PA 19140, USA; 2 Department of Cardiology, New York Presbyterian Hospital, Columbia University Medical Center, New York, NY 10032, USA; 3 Department of Biomedical Education and Data Science, Center for Biostatistics and Epidemiology, Lewi Katz School of Medicine, Temple University, Philadelphia, PA 19140, USA

**Keywords:** chronic thromboembolic pulmonary hypertension, echocardiogram, pulmonary hypertension, right ventricular dysfunction, right ventricular failure

## Abstract

**Background:** Pulmonary thromboendarterectomy (PTE) remains the preferred treatment for surgical accessible thrombus in patients with chronic thromboembolic pulmonary hypertension (CTEPH). However, residual pulmonary hypertension (PH) can persist post-PTE. **Methods:** A retrospective single-center analysis of patients that underwent PTE between 2013 and 2023. At 3-month follow-up, we performed a qualitative Echo-Doppler (DE) assessment and applied a semi-quantitative DE scoring system (DESS), assigning point values for six DE parameters: right ventricle (RV) size, RV shape (systolic base–apex ratio), RV function, septal position, tricuspid regurgitation (TR) and RV outflow tract notching (RVOTN). Higher scores suggested a more significant residual PH syndrome. **Results:** A total of 188 subjects (80%) did not require further PH intervention at ≥3 months (Group A); 48 (20%) required ongoing PH treatment (Group B). The pre-PTE median DESS was 10 and the post-PTE median DESS was 3.00 (range 0–16). The maximum DESS was 17. Using ROC analysis, post-PTE DESS strongly discriminated between Group A and B (AUC 0.76; 95% CI 0.65–0.89; *p* < 0.001). A post-PTE DESS of >6.5 differentiated Group A and B. Evidence of TR (OR 0.191, CI 0.103–0.279; *p* < 0.0001) and RV enlargement (OR 0.242; CI 0.153–0.330; *p* < 0.0001) at follow-up was associated with a need for additional PH interventions. **Conclusions:** Serial DE examination is a viable, noninvasive method to assess significant residual PH post-PTE.

## 1. Introduction

Chronic thromboembolic pulmonary hypertension (CTEPH) is a life-threatening disease that is caused by obstruction of the pulmonary arteries (PAs) by single or recurrent pulmonary thromboemboli that do not undergo complete resolution after at least 3 months of anticoagulation therapy, and consequently precipitate pulmonary hypertension (PH) and right heart failure [1]. Up to 4% of acute pulmonary embolism cases may progress to symptomatic CTEPH [2]. CTEPH is a rare disease, which has led to lack of physicians’ awareness and difficulties in timely diagnosis and management. Patients present unexplained dyspnea or signs of right sided heart failure. Other symptoms include chest pain, cough, and hemoptysis. A ventilation–perfusion lung scan is frequently used as a screening modality to rule out CTEPH. This will prompt other multimodality imaging tools to confirm the presence, extent, distribution and severity of chronic thromboembolic pulmonary disease, such as computed tomography pulmonary angiography, catheter-based pulmonary angiography with digital subtraction, dual-energy computed tomography and/or magnetic resonance imaging.

Pulmonary thromboendarterectomy (PTE) is the treatment of choice for patients with operable CTEPH as it is potentially curative and surgical mortality rates are low, particularly when carried out at expert centers. Pulmonary vascular obstructions in the main, lobar and segmental pulmonary arteries are usually accessible for surgical removal and considered operable. In contrast, diseases located in subsegmental arteries can be more technically difficult, but are still considered surgical cases at PTE expert centers. Rates of PTE have steadily increased over the past decade, with around 0.9 PTE procedures per million population being performed annually in the USA, and around 1.7 PTE procedures per million population being performed annually in Europe [3,4,5]. The first international CTEPH registry [6] found that one third of CTEPH patients were considered inoperable, and had less favorable 1-year outcomes overall than patients undergoing PTE surgery. Although PA pressure and vascular resistance values can normalize post-PTE, a significant minority of patients demonstrate residual PH due to surgically inaccessible chronic thromboemboli or distal pulmonary vasculopathy. Multimodal evaluation by multidisciplinary teams will improve diagnosis and management of patients with CTEPH as it will help establish the operability and surgical candidacy of the patients.

Right heart catheterization (RHC) is the standard method for the diagnosis of residual PH after PTE. Previous studies have used various definitions, including mean PA pressure (mPA) > 25–35 mmHg and pulmonary vascular resistance (PVR) > 240–500 dynes/sec/cm^5^, for the diagnosis of residual PH. The incidence ranges anywhere between 15% and 51% based on the definition used [7,8,9,10]. Newer definitions of PH have also introduced the possibility of classifying individuals with PH when mPA > 20 mmHg [11]. However, it is not clear whether all subjects should be subjected to RHC following PTE, particularly those who are asymptomatic and possess no clinical, biomarker or cardiac imaging findings to suggest clinically significant residual PH.

Echocardiography is a noninvasive, widely available, inexpensive technology that has been shown to reliably demonstrate 2-D and Doppler findings related to elevated PA pressure, mean right atrial pressure, severity of tricuspid regurgitation, PVR and right heart function [12,13]. RV function assessment is an essential part of the initial and serial evaluation of CTEPH. In the preoperative echocardiographic evaluation of patients with CTEPH, RV dysfunction is described by decreased tricuspid annular plane systolic excursion (TAPSE, <18 mm), RV fractional area change (FAC, <35%) and tricuspid annulus velocity (s’ < 9.5 cm/s) [14,15]. However, evidence to support the use of Echo-Doppler (DE) characteristics to assist in identifying residual PH in post-PTE patients is lacking. Herein, we report on the DE characteristics at 3 months post-PTE associated with residual PH that require further medical and/or procedural intervention.

## 2. Materials and Methods

### 2.1. Study Population

**Figure 1:**
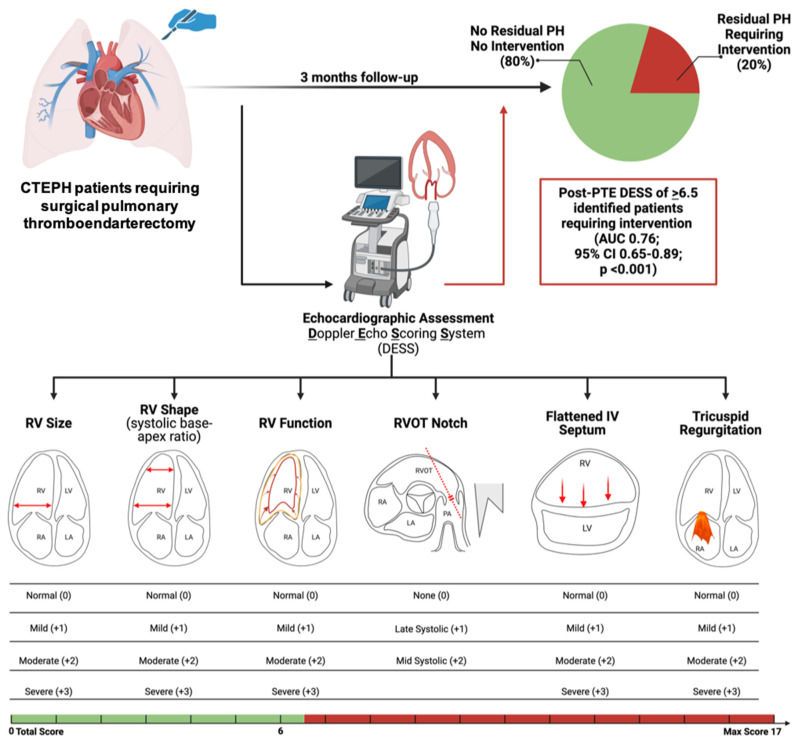
Flow chart showing study design and DESS. Created with Biorender.

We conducted a retrospective single-center analysis of all CTEPH patients who underwent PTE between July 2013 and 2023 at Temple University Hospital. All patients who had 3-month post-PTE follow-up visit data along with DE data and RHC were included. We excluded echocardiograms that did not have the 6 parameters required for the score. Patients were classified into 2 groups: Group A—patients who underwent detailed post-PTE assessment and were deemed to not require any additional PH intervention (PH medical therapy, balloon pulmonary angioplasty or redo PTE); Group B—patients who required PH intervention (Figure 1). Further medical or procedural intervention was defined as the introduction of PH medical therapy, balloon pulmonary angioplasty, or a PTE redo either in isolation or in combination. Therapy was tailored based on a multidisciplinary team evaluation with careful review of appropriate strategies based on the treatment response after surgery.

### 2.2. Hemodynamic Data

In all the patients included in this study, hemodynamic data was obtained with an RHC in the preoperative setting and with a continuous PA catheter monitor in the immediate postoperative period. Right atrial pressure (RAP), PA pressures, pulmonary capillary wedge pressure (PCWP), total pulmonary resistance (TPR), PVR, cardiac output (CO) and cardiac index (CI) were measured. In the immediate post-op period, PCWP was not routinely obtained as a safety precaution after a recent endarterectomy.

### 2.3. Transthoracic Echocardiography

Echocardiography was performed after 3 months post-PTE. All echocardiograms were performed by experienced sonographers. All examinations were made in accordance with the American Society of Echocardiography (ASE) recommendations [16]. The ASE evaluated the right ventricle with measurements of the major and minor axes, TAPSE and tricuspid annular systolic velocity.

**Figure 2:**
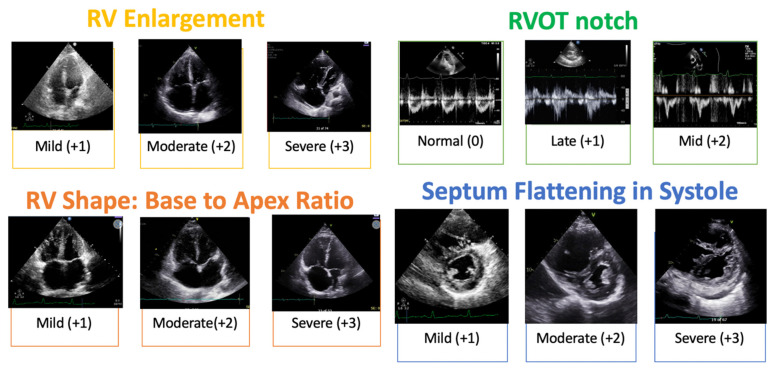
Pictorial examples of DESS. Abbreviations: RV—right ventricle; RVOT—right ventricular outflow tract; TV tricuspid valve.

Two providers (AJ and EO), blinded to preoperative and postoperative clinical information, independently performed qualitative DE assessments and applied a semi-quantitative DE scoring system (DESS), assigning point values for 6 parameters: RV size, RV shape (systolic base-to-apex ratio) [17], RV systolic function, septal position in systole, degree of tricuspid regurgitation (TR) and RV outflow tract (RVOTN) Doppler notching [18] (Figure 2). The shape of the RV was established by the degree of tapering of the RV diameter from base (just above the tricuspid annulus) to apex (level of the moderator band) in the apical four-chamber view [17]. The RV outflow tract Doppler notching in the mid-systolic notch was characterized by a distinct notch or nadir within the initial 2/3 of the systolic ejection period, dividing the flow pattern into two peaks [18]. The late systolic notch pattern was defined by transient flow velocity deceleration or notching on the terminal aspect of the Doppler signal [18]. Inter-observer reproducibility was assessed using a randomly selected subset of 44 patients. A second observer repeated the same measurements to obtain the inter-observer reproducibility. Intra-class correlation coefficients (ICC) between the measurements were used to assess inter-observer reproducibility. An ICC > 0.8 was considered to be almost perfect agreement.

### 2.4. Statistical Analysis

Descriptive data for continuous variables are presented as means ± SD or as medians (IQR) when appropriate. Categorical data was compared using Fisher’s exact test. Comparisons between groups for continuous variables were performed using unpaired two-sample t tests or the Mann–Whitney test, as appropriate. Analysis of group effects with repeated exercise measures was performed by comparing mean slope coefficients from individual linear regressions. Intra-class correlation coefficients (ICCs) between the measurements were used to assess the inter-observer reproducibility. A *p*-value of <0.05 was considered significant. Data were analyzed using IBM SPSS statistics V.22 software (SPSS Inc., Chicago, IL, USA) and Stata 17.0 (StataCorp LLC., College Station, TX, USA).

## 3. Results

### 3.1. Baseline Characteristics Before Surgery

We collected data from July 2013 and 2023, in which 393 patients underwent PTE at Temple University Hospital. We included 236 patients who had 3-month post-PTE follow-up visit data and complete DE data. Patients were excluded if their echocardiogram was missing all six parameters required for DESS (*n* = 157) (Figure 1). Group A included 188 patients (80%) who did not require any additional PH intervention post-PTE; Group B included 48 patients (20%) requiring PH intervention post-PTE (27 patients required only medical therapy, 19 patients required BPA and medical therapy, and 2 required repeat surgery). Baseline characteristics and comorbidities of the patients are shown in Table 1. The cohort was characterized by a mean age of 59.5 ± 14.7 years old, 115 (48.7%) were female, 126 (53%) were White and 78 (33.1%) were Black. Mean BMI was 31.9 ± 7.9 kg/m^2^. The majority of the patients had pre-surgical World Health Organization (WHO) functional class of II (27.1%) or III (52.5%) symptoms, and the mean pre-PTE 6 min walk distance (6MWD) was 339.2 ± 136.2 m. Pre-PTE, 77 (32%) patients were on PH medical therapy.

### 3.2. Hemodynamic Data

RHC data at baseline and in early follow-up (≥48 h after PTE) for the overall cohort is shown in Table 2. At baseline, the mean PA pressure was 43.8 ± 11.7 mmHg, PVR was 8.4 ± 4.4 WU, TPR was 11.1 ± 4.8 WU and CI was 2.1 ± 0.5 L/min/m^2^. Post-PTE, there was a 46% reduction in mean PA pressure, an increase of 22% in CI and a 59% decrease in TPR.

### 3.3. Echocardiographic Data

**Figure 3:**
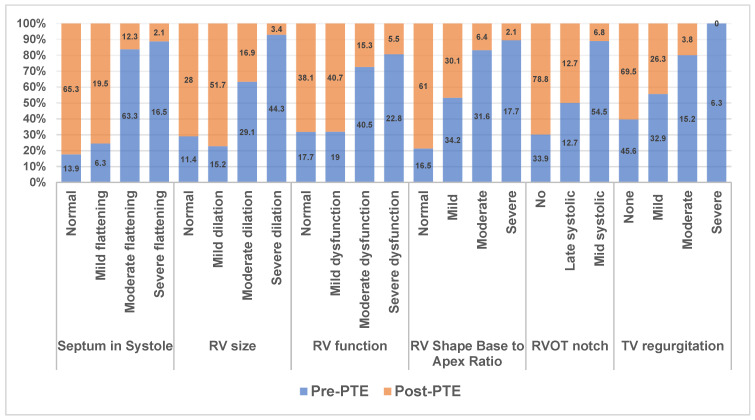
Echo-Doppler scoring system (DESS) at baseline and at 3-month post-PTE follow-up.

DE assessment at 3 months post-PTE showed dramatic improvements for septal position, RV size, RV function, RV shape, right heart afterload (RVOT notching) and the degree of TR (Figure 3). The vast majority of post-PTE subjects had either normal/mildly abnormal findings for the following: RV size (79.4%), RV function (79.4%), RV shape (91.1%), septal position (84.8%), degree of TR (95.3%) and no RVOT notching (78.8%). Hence, the pre-PTE median DESS was 10 and the post-PTE median DESS was 3.00 (range 0–16). The maximum DESS was 17.

**Figure 4:**
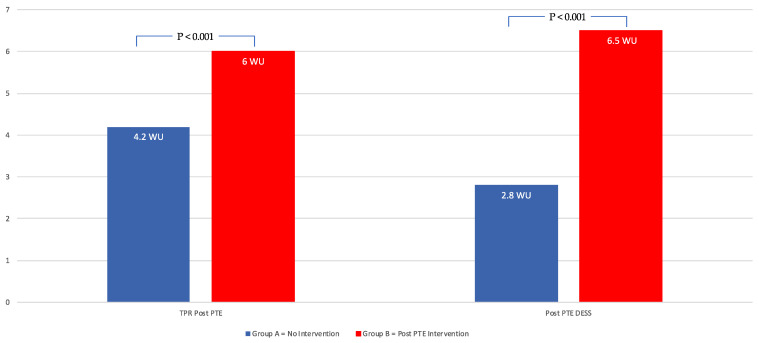
Differences in TPR and DESS in patients post-PTE requiring and not requiring additional intervention.

When comparing the subjects that required additional intervention (Group B) and those that did not (Group A), there were no significant differences in age, BMI, comorbidities or pre-PTE hemodynamics (Table 3). Group B subjects had a higher TPR (6 ± 2.5 vs. 4.2 ± 1.6 mmHg; *p* < 0.001) and DESS (6.5 ± 4.6 vs. 2.8 ± 2.5; *p* < 0.001) (Figure 4).

**Figure 5:**
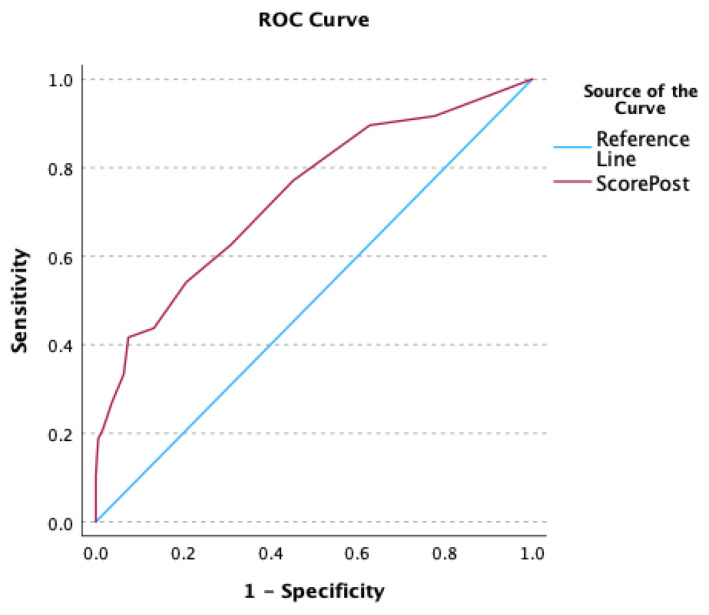
ROC curve analysis of DESS to predict need for additional PH therapy post-PTE.

Using ROC analysis, post-PTE DESS was found to have a strong ability to differentiate between Group A and B (Figure 5, AUC 0.76; 95% CI 0.65–0.89; *p* < 0.001) with a post-PTE DESS of 6.5 or more. A post-PTE DESS of 6.5 or more has an odds ratio of 8.5 (95% CI 3.8–18) of requiring further intervention post-PTE. To meet the DESS threshold of <6 requires no more than mild abnormalities for all six DE parameters. Evidence of tricuspid valve regurgitation (OR 0.191, CI 0.103–0.279; *p* < 0.0001) and RV enlargement (OR 0.242; CI 0.153–0.330; *p* < 0.0001) at follow-up were highly associated with a need for additional PH interventions post-PTE.

### 3.4. Inter-Observer Variability Agreement

Inter-observer variability agreement was assessed using a randomly selected subset of 44 patients. A second observer repeated the same measurements to obtain the inter-observer reproducibility. Intra-class correlation coefficients (ICCs) between the measurements were used to assess inter-observer reproducibility. An ICC > 0.8 was considered to be almost perfect agreement. The ICC for inter-observer reproducibility was 0.87 (95% CI 0.759–0.928).

## 4. Discussion

In the current study, we report on a semi-quantitative DE assessment system, used to differentiate patients following PTE surgery who did not require further PH intervention from those with residual PH requiring further medical and/or procedural intervention. Post-PTE, 80% of subjects did not require further PH intervention post-PTE (Group A) versus the other 20% (Group B) of the patients, who did receive medical and/or procedural intervention post-PTE. The median DESS dropped from 10 to 3.0 post-PTE. The group requiring intervention had a higher DESS post-PTE compared to the no-intervention group, 6.5 versus 2.8. When the DESS score was >6.5, patients were 8.5 times more likely to require post-PTE intervention. This study is the first to demonstrate the use of DE examination to differentiate the presence versus absence of clinically significant residual PH following PTE.

Following PTE, there is no consensus approach on how residual PH should be identified or defined. For patients with unfavorable outcomes due to persistent right heart failure or significant residual PH, consideration for additional interventions will be required. A trial of medical therapy using riociguat or other pulmonary vasodilators can be entertained, as well as a redo of PTE or BPA. Given that the majority of patients have PTE performed at CTEPH centers of excellence, with a dramatic clinical improvement with normal or near-normal hemodynamics and right heart function routine ‘screening’, RHC post-PTE may not be justified. Therefore, the use of echocardiography for evaluation of residual PH can be used as an effective screening tool.

Residual PH is associated with high perioperative mortality and morbidity [19]. Postoperative PVR predicts in-hospital and 1-year mortality [19]. Freed et al. [8] showed that residual PH causes symptoms and limited functional capacity. The 6MWD has been described to be significantly lower post-PTE in individuals that do not achieve normal hemodynamics [20].

Despite recent changes in the definitions of abnormal mPAP and PVR [11], there remains no standardized definition of what constitutes clinically significant residual PH following PTE. This is due in part to the fact that current hemodynamic cutoffs do not account for an individual patient’s baseline values, the magnitude of change from pre-PTE measurements or the relationship between pulmonary pressures, PVR and right heart function—specifically, RV-PA coupling. The RV-PA coupling relationship is the central hemodynamic perturbation in any form of precapillary PH and undergoes dynamic remodeling in the months following PTE. Growing evidence suggests that preserved RV-PA coupling, and therefore compensated right heart function, may be more prognostically meaningful than elevated pulmonary pressures in isolation. This may explain the observation that although 51% of patients met the residual PH definition of mPAP > 25 mmHg, only 30% were initiated on PH medical therapy. Similarly, it may clarify why much higher thresholds—mPAP > 38 mmHg and PVR > 5.3 mmHg/L/min—were required to identify a cohort at increased risk of death post-PTE [10]. These patients were likely able to maintain a compensated right heart despite these hemodynamic perturbations.

In our practice, we use the 3-month post-PTE DE examination as a key part of the postoperative assessment, integrated with clinical, functional, imaging and biomarker evidence, in order to determine who is appropriate for further investigation via RHC. Transthoracic echocardiograms provide insight into the pathophysiology of CTEPH. Using this approach, hemodynamic data is paired with their DE data, which allows contextualization of the hemodynamic information to gain better insight into the degree of RV-PA coupling. The DESS represents a comprehensive and simple-to-use DE scoring system, designed to capture parameters reflecting RV size, RV shape, septal position during systole, RV function, TR severity and RV afterload. Using ROC analysis, a post-PTE DESS of 6.5 (AUC 0.74; *p* < 0.001) was found to have a strong ability to differentiate between Group A and B (Figure 5). Importantly, to meet the DESS threshold of <6 required no more than mild abnormalities for all six DE parameters.

High pulmonary vascular resistance in CTEPH leads to RV dilation, right-to-left interactions (septal flattening) and RV systolic dysfunction [11]. RV shape (ratio of RV base to RV apex less than 1.5) and RV notching (mid or late) have also been shown to be associated with elevated pulmonary vascular resistance [17,18,21]. Restoration of blood flow to previously occluded segments results in an immediate reduction in PVR and increase in CO [22,23], and these were similarly demonstrated in this study. A corresponding improvement in the abovementioned echocardiographic variables was shown in multiple studies in the past [24,25]. We found similar results in our study with the majority of patients at 3 months post-PTE.

RV fractional area change, RV free wall strain and RV Tei index have also been shown to be useful parameters that improve post-PTE [26,27,28,29]. But these are time consuming and difficult to perform. Although TAPSE is simple to use and shown to be useful in evaluating RV function in PAH patients, it is shown to be a poor metric of RV function post-PTE in multiple studies [26,27]. TAPSE predominantly reflects longitudinal RV contraction. Pericardiectomy during PTE redistributes RV contraction from a predominantly longitudinal to a more radial pattern, resulting in lower TAPSE values despite preserved RV function. Typical two-dimensional echocardiogram findings in CTEPH patients include right ventricular enlargement and hypertrophy, depressed right ventricular function, dilation of the inferior vena cava (to estimate elevated mean right atrial pressure) and flattening of the interventricular septum in systole (estimating pressure overload) and in diastole (estimating volume overload). In cases with septal flattening, there will also be changes in the left ventricular eccentricity index, which will signal issues like pressure overload. The degree of tricuspid valve regurgitation can be estimated by color Doppler, and the peak systolic velocity of the tricuspid regurgitant jet can be measured by continuous-wave spectral Doppler imaging. Acknowledging the geometric limitations of the RV and the difficulties in obtaining accurate parameters is important, as these emphasize the importance of using a combination of markers to provide an accurate assessment.

Evidence of tricuspid valve regurgitation by color Doppler (OR 0.191, CI 0.103–0.279; *p* < 0.0001) and RV enlargement (OR 0.242; CI 0.153–0.330; *p* < 0.0001) at follow-up was highly associated with the need for additional PH interventions post-PTE. Other studies [30] on PTE patients have demonstrated that in patients with proximal thromboembolic disease (types 1 and 2), there is a significant improvement in PASP, PVR and tricuspid regurgitation after surgery compared with patients with segmental artery and distal small vessel vasculopathy (type 3–4 disease). Duration of preoperative pulmonary hypertensive symptoms, length of time with diagnosed tricuspid regurgitation and severity of tricuspid regurgitation were not predictive of postoperative tricuspid valvular recovery.

Our scoring system is simple and easy to integrate into everyday clinical practice. The echo variables we used were either (1) already obtained and reported in routine practice or (2) quickly assessed from universally acquired images. DESS can be used to identify residual PH post-PTE and to monitor treatment efficacy during serial follow-up. This can potentially be used as an easy screening tool in algorithmic management in the follow-up of patients with CTEPH who undergo PTE. In addition to screening, it can help to assess prognosis and monitor disease stability.

## 5. Limitations

One potential limitation of this study is its single-center retrospective design. CTEPH is a rare disease, and few centers have the expertise to perform PTE. Our sample is similar to or higher than prior studies. Prospective multi-center studies would allow further validation of the post-PTE DESS. The proposed post-PTE DESS is based on echocardiographic evaluation at 3 months post-surgery, which has inherent limitations such as the quality of acoustic windows, intra- and inter-observer variability, operator dependence, and the complex geometry of the RV. However, the strong discriminatory ability of the DESS along with its ICC of 0.87 suggests that post-PTE DESS can be implemented in spite of these potential limitations. We focused on echocardiographic variables commonly obtained in clinical practice. Additionally, including other quantitative parameters such as RV strain, RV Tei index, RV FAC or advanced techniques such as 3D echo, CT and MRI might provide more comprehensive information. However, these parameters and techniques are significantly more time- and resource-intensive, which would limit their clinical applicability. We used echocardiogram data at 3 months post-PTE, but we have yet to determine the appropriate timing to apply post-PTE DESS. When we used post-PTE DESS at 1 week post-PTE and then at 3 months PTE, we saw serial improvements over time in scores that were attributed to pulmonary vascular remodeling related to the removal of the pulmonary artery obstruction. Finally, we did not include in the scoring system biomarker data (e.g., brain natriuretic peptide) or clinical data (e.g., 6MWD), but this may help in future validation when implementing this tool in clinical practice.

## 6. Conclusions

To our knowledge, this is the first study to demonstrate the utility of noninvasive Echo-Doppler parameters in differentiating the presence or absence of residual PH at 3 months post-PTE deemed to require treatment intervention. A post-PTE DESS of >6.5 suggests clinically significant residual PH requiring treatment.

## Figures and Tables

**Table 1:** Baseline characteristics and comorbidities before surgical pulmonary thromboendarterectomy.

Variables	Mean ± SD
Age (years)	59.5 ± 14.7
BMI (kg/m^2^)	31.9 ± 7.9
6 min walk distance (meters)	339.2 ± 136.2
Variables	*n* (%)
Race/Ethnicity	
White	126 (53.4%)
Latinx	15 (6.4%)
Black	78 (33.1%)
WHO Functional Class	
I	18 (7.6%)
II	64 (27.1%)
III	124 (52.5%)
IV	21 (8.9%)
Pre-PH therapy	77 (32%)
DOAC	106 (44.9%)
Tobacco use	104 (44%)
History of thromboembolic events	189 (80.1%)
History of DVT	124 (53%)

Abbreviations: DOAC—direct oral anticoagulant; DVT—deep vein thrombosis; PH—pulmonary hypertension; WHO—World Health Organization.

**Table 2:** Right heart catheterization data at baseline and early post-pulmonary thromboendarterectomy follow-up.

RHC Parameters	Baseline Hemodynamics Mean ± SD	Early Post-PTE Hemodynamics Mean ± SD	*p* Value
RAP (mmHg)	10.2 ± 5	7.9 ± 3.7	<0.001
Systolic PA (mmHg)	73.9 ± 20.7	39.8 ± 15.2	<0.001
Diastolic PA (mmHg)	26.3 ± 8.2	15.9 ± 6.3	<0.001
Mean PA (mmHg)	43.8 ± 11.7	23.9 ± 8.5	<0.001
PCWP (mmHg)	11.9 ± 5.1	NA	
CO (lpm)	4.4 ± 1.2	5.6 ± 1.3	<0.001
CI (lpm/m^2^)	2.1 ± 0.5	2.7 ± 0.5	<0.001
TPR (WU)	11.1 ± 4.8	4.5 ± 1.9	<0.001
PVR (WU)	8.4 ± 4.4		

Abbreviations: CI—cardiac index; CO—cardiac output; NA—not available; PA—pulmonary artery; PCWP—pulmonary capillary wedge pressure; PTE—pulmonary thromboendarterectomy; PVR—pulmonary vascular resistance; RAP—right atrial pressure; RHC—right heart catheterization; SD—standard deviation; TPR—total pulmonary resistance; WU—Wood Units.

**Table 3:** Comparing pre- and post-PTE demographic, hemodynamic and echo parameters between Group A and Group B.

Variables	Group A No Intervention	Group B Intervention	*p* Value
Age (years)	58 ± 14	61 ± 14	0.204
BMI (kg/m^2^)	32.1 ± 7.9	30.5 ± 8	0.218
Pre-PTE Mean PA pressure (mmHg)	43 ± 12	48 ± 10	0.357
CI (L/min/m^2^)	2.2 ± 0.5	1.9 ± 0.5	0.580
TPR (WU)	10.5 ± 4.8	13.3 ± 4.8	0.177
Mean DESS	9.1 ± 4.3	9.2 ± 4.8	0.9
Post-PTE Mean PA pressure (mmHg)	22.4 ± 7.7	29.9 ± 9.04	0.421
CI (L/min/m^2^)	2.7 ± 0.5	2.7 ± 0.5	0.457
TPR (WU)	4.2 ± 1.6	6 ± 2.5	<0.001
Mean DESS	2.8 ± 2.5	6.5 ± 4.6	<0.001

Abbreviations: BMI—body mass index; CI—cardiac index; DESS—Doppler echocardiogram scoring system; PA—pulmonary artery; PTE—pulmonary thromboendarterectomy; SD—standard deviation; TPR—total pulmonary resistance; WU—Wood Units.

## Data Availability

Data may be shared upon request at the discretion of the corresponding author.
